# Design of a chimaeric antigen and its use in the detection of IgG antibodies against rubella virus

**DOI:** 10.1186/s12985-022-01760-y

**Published:** 2022-02-23

**Authors:** Wenyue Xing, Li Li, Jingnan Zhang, Chunli Ma, Xin Xue, Shumei Ye, Huiting Xue, Ruiping Hu, Yanhua Ma, Hong Yuan

**Affiliations:** 1 College of Basic Medicine, Inner Mongolia Medical University, Hohhot, 010110 Inner Mongolia China; 2 The People’s Hospital of Jiang County, Shanxi, 043600 China; 3 School of Life Sciences, Inner Mongolia University, Hohhot, China

**Keywords:** Rubella virus, Prokaryotic expression, Serological diagnosis, Chromatography purification

## Abstract

**Background:**

Rubella virus (RV) is the causative agent of rubella or German measles. Although most infections cause only mild self-limited measles-like illness, the infection in pregnant women can cause severe foetal malformation or even miscarriage, especially in the first 3 months of pregnancy. Therefore, it is of great practical significance to establish a simple and sensitive RV detection method.

**Methods:**

The partial epitopes of the E1 and E2 proteins from Rubella Virus were selected as the target sites, the sequence of the selected antigenic sites of the E1 and E2 were linked by a linker. The expression plasmid P6T was constructed by inserting the gene into PET-32A + with a histidine Tag. The P6 protein was induced and expressed in *Escherichia coli* L21 (DE3) and purified by nickel column affinity. The protein P6 antigen was identified by Western blotting analysis, and an anti-P6 antibody ELISA was established to test known serum samples to evaluate the capability of this method.

**Results:**

After purification, the concentration and purity of the protein P6 were 0.283 mg/mL and more than 80%, respectively. Western blotting analysis showed that the protein P6 could react with rubella virus positive serum. By ELISA, 36 negative sera and 58 positive sera were detected. The coincidence rate, specificity and sensitivity of the ELISA were 86.2%, 88.89% and 84.48%, respectively. The P6 ELISA with a kappa coefficient of 0.715, *P* < 0.05, indicated excellent consistency.

**Conclusions:**

The protein P6 with excellent antigenicity obtained from prokaryotic expression followed by chromatography purification could prove useful for early diagnosis of RV infection.

## Background

Rubella virus (RV) is the causative agent of rubella or German measles [1]. Although most infections cause only mild self-limited measles-like illness, the infection in pregnant women can cause severe foetal malformation or even miscarriage, especially in the first 3 months of pregnancy [2, 3]. Therefore, it is of great practical significance to establish a simple and sensitive RV detection method. After Rubella virus infection, IgM appears earlier than IgG. However, it is maintained for a short period of time and decreases in expression after 1 month until it turns negative. IgG antibody appears after IgM, and the level of IgG antibody shows a rising trend. The peak IgG expression appears 1–2 months after infection. Its level decreases very slowly. IgG can remain in the body for long periods of time, even up to a few decades [4–6]. Therefore, IgM can be used as a marker for early pathogen infection, and IgG antibody can be used as a marker for late infection and after infection.

RV has three structural proteins: one capsid protein (C) and two envelope proteins (E2 and E1). C is rich in arginine and proline residues, which make it positively charged and facilitate its reaction with genomic RNA during nucleocapsid formation [7]. As type I membrane proteins, E1 and E2 are heterodimerized to form complexes on the surface of the virus [8]. The main function of the spinous process complex is to collect host cell receptors and to mediate the fusion between the virus and host cell membrane [9]. For RV IgG antibodies, E1 has the most epitopes compared to E2 and C protein [10]. It has been found that a protein expressed by serially linking different epitopes of the virus can be used for the detection of RV-specific IgG with good diagnostic efficacy [11]. In this paper, the immunodominant regions of two RV structural proteins were expressed in tandem on the same protein, and the recombinant protein was applied to an indirect ELISA for detecting RV-IgG.

## Methods

### Materials and reagents

*Escherichia coli* (*E. coli*) DH5α competent cells were obtained from our own laboratory. BL21 (DE3) competent cells was purchased from TransGen Biotech(China). T4 DNA ligase and all restriction enzymes were purchased from NEB. HRP-labelled goat anti-human IgG was purchased from Santa Cruz (USA). DAB (USA, Vector labs). Samples from 94 umbilical cord serum samples collected in 2014 from HuNan Province by the Chinese Center for Disease Control and Prevention. The origin of samplesis from individuals with history of 2B genotype infection. All of them were confirmed by a RV ELISA Kit (Germany, VirionSerion), of which 58 were positive and 36 were negative.

### Construction of the expression plasmid P6T

Based on references [12–14], sequences with antigenic sites of E1 (199-286) and E2 (1-115) were selected, they were linked by a linker (GGGGSGGGGSGGGGS). After codon optimization, restriction endonuclease sites for BamHI and HindIII were added at both ends. The construct was then synthetized by the Invitrogen company (named P6T). P6T (18 ng/µL) was digested by BamHI and HindIII. P6T was purified and ligated to the pGEM-T expression vector. Then, the plasmid pGEM-T-P6T was transformed into *E. coli* DH5α. The positive clones were further identified with the restriction endonuclease digestion of BamHI and HindIII. The sequence was confirmed by SinoGenoMax Company.

### Preparation of the protein P6

Plasmid P6T was transformed to BL21 (DE3) competent cells. The transformed BL21 (DE3) was inoculated in LB medium containing 50 µg/mL ampicillin and induced expression. After cleaning twice with 10 mL high salt lysate (20 mM Tris, 0.5 m NaCl, 2 mM EDTA, 5% glycerol, 0.5% TritonX-100, PH8.0), ice water bath ultrasound (270 W, ultrasound 5S, pause 25S) for 15 min. After centrifugation, the precipitate was dissolved in 10 mL equilibrium buffer solution (20 mM Tris, 0.5 m NaCl, 10 mM imidazole, PH8.0), and centrifuged again. The resulting supernatant was filtered by a 0.45 um filter. The filtrated supernatant was purified by nickel column and eluted with gradient elution of 60 mmol/L and 300 mmol/L imidazole. The elution samples were analyzed by SDS-PAGE and the content and distribution of the target protein were observed [15]. The eluent with higher purity was desalted by dialysis, the target protein was concentrated by ultrafiltration centrifuge, and stored at 4 ℃ for later use.

### Western blotting analysis of the protein P6

Protein P6 was separated using 10% SDS-PAGE and transferred to PVDF membranes. The membranes were blocked with 5% milk for 1 h at room temperature and were then washed 3 times in PBST. The membranes were then incubated with the cord blood serum at 1:200 dilution with the blocking solution at 4 °C overnight and washed 3 times. The membranes were then incubated with goat anti-human IgG-HRP-linked antibody (America, Santa Cruz Inc, 1:5 000) for 1 h at room temperature and washed 3 times.The protein levels were measured using a DAB Horseradish Peroxidase Color Development Kit.

### The protein P 6 was detected by indirect enzyme-linked immunosorbent assay

After diluting the P6 sample with buffer solution, 100 µl of sample was added into each well, and the sample was coated at 4 °C for 12 h. Then, the plate was washed four times with PBST buffer. Then, 200 µl of blocking solution was added to each well, and the plate was stored at 37 °C for 2 h. The plate was washed with PBST buffer four times. Then, 100 µl of serum diluted with a blocking solution (PBST solution containing 5% BSA) was added. After being stored at 37 °C for 1 h, the plate was washed four times with PBST. Then, 100 µL of goat anti-human antibody (America, Santa Cruz Inc) diluted with blocking solution was added and incubated at 37 °C for 30 min. The plate was washed as described above. Then, 100 µL of TMB (America, KPL) was added to each well. The plate was stored in the dark for 5–15 min at RT. The optical density (OD) value at 450 nm (A450) was measured by microplate reader, and the OD value at 630 nm (A630) was used as a reference. The optimal conditions of serum dilution, antigen concentration and reaction time were established by performing a checkerboard titration. When the ratio of positive serum to negative serum A450s (P/N) was the best critical value, the coated antigen concentration was the best envelope concentration [16, 17].

### Data analysis

Receiver operating characteristic (ROC) curve was drawn according to the OD value measured by the enzyme labelling method. The optimum critical value was determined by the Yoden index (Yoden index = sensitivity + specificity − 1). The sensitivity (Sensitivity = [true positive cases/(true positive cases + false negative cases)] × 100%) and specificity(Specificity = [true negative cases/(true negative cases + false positive cases)] × 100%) of the new detection method were calculated according to the critical value. SPSS18 software was used for statistical analysis of the experimental data.

## Results

### P6 was obtained by prokaryotic expression

**Fig. 1:**
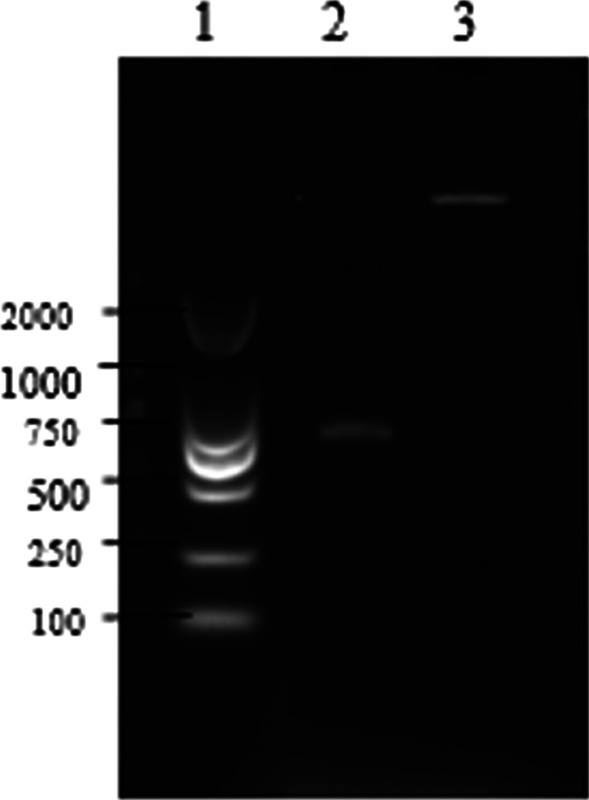
Agarose gel electrophoresis of P6T and its expression vector after double digestion. The target fragment P6T linked to PGEM-T and the expression vector PET-32a (+) were digested with restriction enzymes BamHI and HindIII, and the obtained target fragment was recovered for identification by agron gel electrophoresis. 1: DNA marker, DL2000; 2: P6T plasmid; 3: double restriction enzyme (BamHI, HindIII) digestion product of pET-32a (+)

**Fig. 2:**
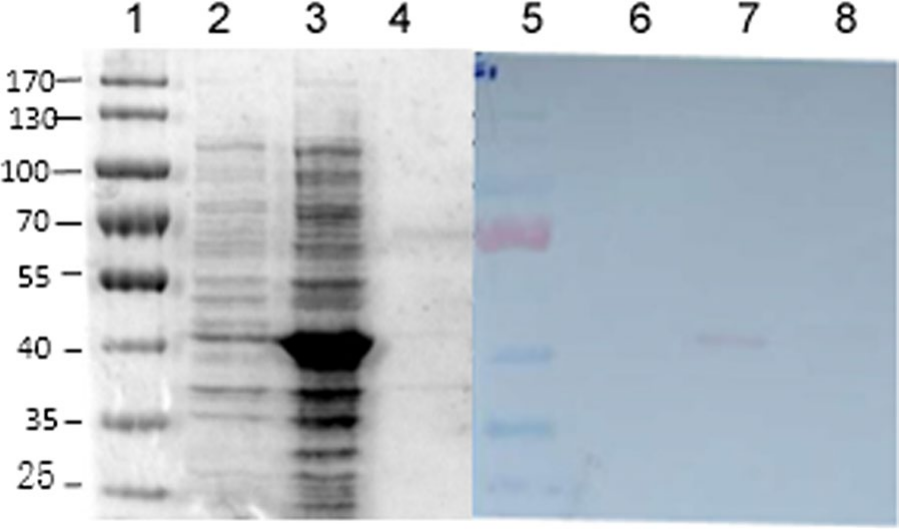
Western blot identification of induced expression of P6 with human serum. The constructed recombinant expression plasmid was transformed into BL21(DE3), and then induced by IPTG to obtain P6 in the form of inclusion body.Western blot was used to identify P6 with positive and negative anti-RV serum respectively, and the desired band was obtained in the positive serum. 1: Marker; 2. control (no IPTG induction); 3. expression product (IPTG induction: 2.5 h, 1 mM IPTG); 4. supernatant of expressed product; 5. marker; 6. control; 7. positive serum + P6; 8. negative serum + P6

The recombinant plasmid with multiple epitopes (named P6T) was constructed by selecting the immunoactive sites on the two structural proteins of RV. The P6T plasmid was approximately 666 bp (Fig. 1). The plasmid was successfully constructed and identified by sequencing and double restriction enzyme digestion (Fig. 1). P6T was expressed in *E. coli,* and the product was named P6. SDS-PAGE showed that the molecular weight of the protein P6 was approximately 44 kDa (Fig. 2). The expected fusion protein expressed encoded by just the pET-32a (+) vector alone would be around 20.4 kDa. In addition, the expected fusion protein expressed encoded by the P6T plasmid would be around 23.29 kDa (GSGLQPRADMAAPPAPPQPPRAHGQHYGHHHHQLPFLGHDGHHGGTLRVGQHHRNASDVLPGHWLQGGWGCYNLSDWHQGTHVCHTKHMDFWCVEHDRPPPATPTPLTTAANATTAAGGGGSGGGGSGGGGSDPGDLVEYIMNYTGNQQSRWGLGSPNCHGPDWASPVCQRHSPDCSRLVGATPERPRLRLVDADDPLLRTAPGPGEVWVTPVIGSQARK). The results showed that the protein P6 could be expressed in *E. coli*. The protein was expressed as inclusion body at 0.283 mg/mL.

Imidazole elution (60 mM, 150 mM, 300 mM) was used for the purification of the target protein. The results of SDS-PAGE showed that P6 protein was the purest with 150 mM imidazole concentration. It is more than 80% pure (Fig. 2).

### The protein P6 reacts with anti-RV serum

The antigenicity of the protein P6 was identified by Western blotting. The results showed that the cord blood serum (RV-positive) as a first antibody and goat anti-human IgG-HRP as a second antibody showed a significant band at approximately 44 kDa. Moreover, RV-negative serum as a primary antibody showed no band (Fig. 2). No protein P6 was added, and no significant bands were observed with either RV-positive or RV-negative serum (Fig. 2). The results showed that the protein P6 could react specifically with RV-positive serum.

### P6 indirect enzyme-linked immunosorbent assay for identification of blood samples

**Fig. 3:**
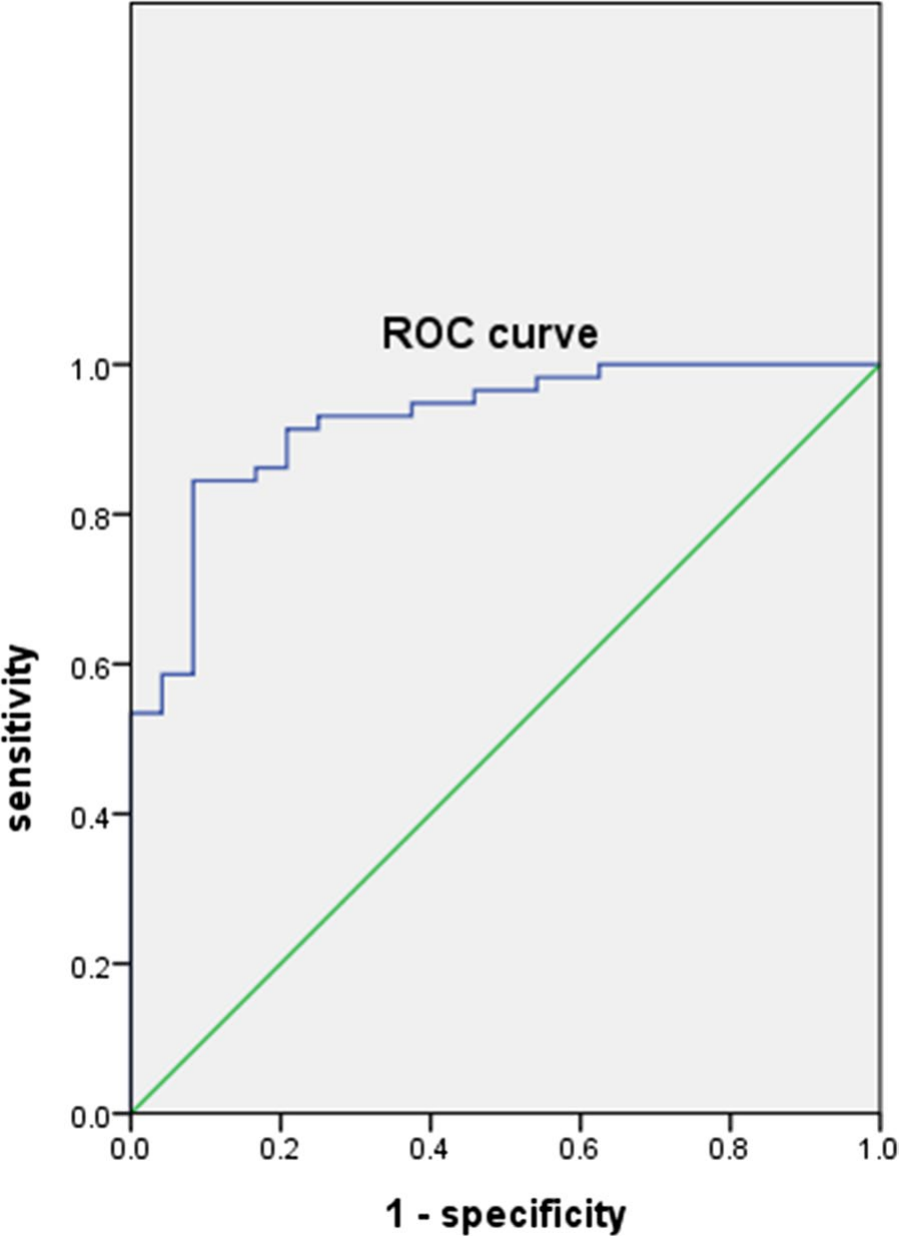
ROC curve of 94 cord blood was detected by P6 indirect ELISA. The optimum critical value was determined by the Yoden index (Yoden index = sensitivity + specificity − 1). Sensitivity = [true positive cases/(true positive cases + false negative cases)] × 100%. Specificity = [true negative cases/(true negative cases + false positive cases)] × 100%. The optimal critical value was 0.317. According to the critical value, the coincidence rate, specificity and sensitivity of the ELISA were 86.2%, 88.89%m 84.48% respectively

The optimal conditions of serum dilution, antigen concentration and reaction time were established. The purified P6 protein was used as antigen at an optimal concentration of 5 µg/ML. The use of purified antigen allowed the testing of sera at a 1:50 dilution without nonspecific reaction. Ninety-four samples of umbilical cord blood (VirionSerion ELISA method) were selected to verify the accuracy of this method (Table 1). An ROC chart was drawn according to the OD value, and the Yoden index was used to determine the best cut off (cut off = 0.317). According to the critical value (Fig. 3), the coincidence rate, specificity and sensitivity of the ELISA were 86.2%, 88.89%, 84.48% respectively.

**Table 1:** The coincidence rate of P6 indirect ELISA and VirionSerion detection

P6 ELISA method	VirionSerion ELISA method		
	Positive	Negative	Total
Positive	49	9	58
Negative	4	32	36
Total	53	41	94

Regarding the Chi-square test results, kappa = 0.715 and *P* < 0.05. The results showed that there was no significant difference between the P6 indirect ELISA and the known results.

## Discussion

There are two types of rubella virus ELISA kits available on the market for testing IgM and IgG antibodies. When pregnant women are infected with RV but not infected in utero, the detection time of IgM antibody is short. When pregnant women are infected with rubella virus in utero, RV IgM antibody can be continuously detected before delivery [18]. Therefore, rubella virus IgM test is suitable for early infection and termination of pregnancy detection. The detection of RV IgG is suitable for the seroepidemiological investigation of RV. The monitoring of RV in China began in 1999 [19]. At present, ELISA is mainly used to investigate the evaluation of individual protective antibodies against RV and the investigation of antibody levels in the population. Meanwhile, the neutralization test is used for auxiliary verification. Because RV is complex to operate and have the risk of infection. At present, the quality of rubella IgG kits produced in China are uneven and the imported kits are expensive [20, 21]. Therefore, it is very important to establish a good rapid clinic serological detecting method for RV.

In this study, *Escherichia coli* BL21 (DE3) was selected as the expression system. Prokaryotic expression system is easy to survive, convenient to purchase and low cost. Its genomic information is simple and clear. The method is simple and efficient for the transformation of recombinant plasmid. When used to express foreign protein, the expression amount is high, and the expressed product is stable. Compared with prokaryotic expression system, Eukaryotic expression system has high cost, complex operation and low yield.

In this paper, the critical value of RV-specific IgG was determined by the ROC curve method. At present, there are many methods to determine the critical value of ELISA, such as the standard curve method, mean plus minus standard deviation method, ROC curve method, response surface optimization method, and uniform design [15, 22, 23]. The ROC curve method is a widely used method to determine the critical value considering specificity and sensitivity [24–26]. The practicality of the proposed method was tested by qualitative analysis of serum data, so the ROC curve method was used to determine the critical value of the proposed method.

There may be some deficiencies in the establishment of the ELISA in the experiment. First, the optimization of ELISA experimental processes involves a number of conditions. Not all the conditions have been tried. Second, only the effective antigen sites of rubella virus E1 and E2 were expressed in this study. Within contrast to the whole virus, the other antigen sites were not expressed. Therefore, there was the possibility of missed detection. Finally, this experiment selected a limited number of serum samples. In order to evaluate the validity of established methods more rigorously, larger sample sizes are required for validation.

## Conclusions

In conclusion, the recombinant protein P6 was expressed by a prokaryotic expression system. An indirect ELISA method for the detection of IgG antibody in serum was established by using P6. Although this method had some shortcomings, it had strong specificity. After optimization, it would be expected to provide a laboratory basis for the development of an RV detection kit with high specificity and sensitivity. The results provide a practical and effective plan for epidemiological surveys of RV in China.

## Data Availability

The datasets used or analysed during the current study are available from the corresponding author on reasonable request.

## Abbreviations

RV: Rubella virus
*E. coli*: *Escherichia coli*
LB: Luria–Bertani
OD: Optical density
